# Recent Advances of Deep Learning in Bioinformatics and Computational Biology

**DOI:** 10.3389/fgene.2019.00214

**Published:** 2019-03-26

**Authors:** Binhua Tang, Zixiang Pan, Kang Yin, Asif Khateeb

**Affiliations:** ^1^Epigenetics & Function Group, Hohai University, Nanjing, China; ^2^School of Public Health, Shanghai Jiao Tong University, Shanghai, China

**Keywords:** computational biology, bioinformatics, application, algorithm, deep learning

## Abstract

Extracting inherent valuable knowledge from omics big data remains as a daunting problem in bioinformatics and computational biology. Deep learning, as an emerging branch from machine learning, has exhibited unprecedented performance in quite a few applications from academia and industry. We highlight the difference and similarity in widely utilized models in deep learning studies, through discussing their basic structures, and reviewing diverse applications and disadvantages. We anticipate the work can serve as a meaningful perspective for further development of its theory, algorithm and application in bioinformatic and computational biology.

## Introduction

Deep learning is the emerging generation of the artificial intelligence techniques, specifically in machine learning. The earliest artificial intelligence was firstly implemented on hardware system in the 1950s. The newer concept with the more systematic theorems, named machine learning, appeared in the 1960s. And its newly-evolved branch, deep learning, was first brought up around the 2000s, and soon led to rapid applications in different fields, due to its unprecedented prediction performance on big data (Hinton and Salakhutdinov, 2006; LeCun et al., 2015; Nussinov, 2015).

The basic concepts and models in deep learning have derived from the artificial neural network, which mimic human brain's activity pattern to intelligentize the algorithms and save tedious human labor (Mnih et al., 2015; Schmidhuber, 2015; Mamoshina et al., 2016). Although deep learning is an emerging subfield recently from machine learning, it has immense utilizations spreading from machine vision, voice, and signal processing, sequence and text prediction, and computational biology topics, altogether shaping the productive AI fields (Bengio and LeCun, 2007; Alipanahi et al., 2015; Libbrecht and Noble, 2015; Zhang et al., 2016; Esteva et al., 2017; Ching et al., 2018). Deep learning has several implementation models as artificial neural network, deep structured learning, and hierarchical learning, which commonly apply a class of structured networks to infer the quantitative properties between responses and causes within a group of data (Ditzler et al., 2015; Liang et al., 2015; Xu J. et al., 2016; Giorgi and Bader, 2018).

The subsequent paragraphs mainly summarize the essential concepts and recent applications of deep learning, together highlight the key achievements and future directions of deep learning, especially from the perspectives of bioinformatics and computational biology.

## Essential Concepts in Deep Neural Network

### Basic Structure of Neural Network

**Figure 1:**
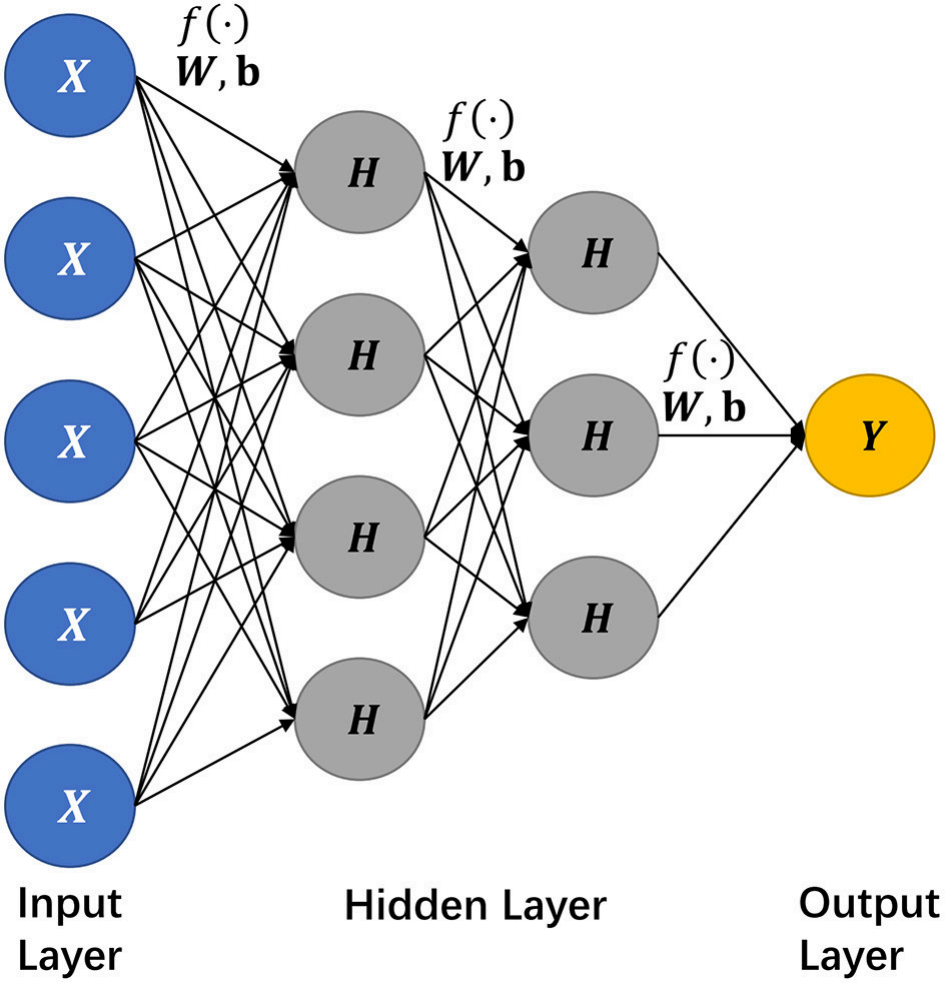
The network structure of a deep learning model. Here we select a network structure with two hidden layers as an illustration, where *X* nodes constitute the input layer, *H*s for the hidden layers, *Y* for the output layer, and *f* (·) denotes an activation function.

Neural network is a class of information processing modules, frequently utilized in machine learning. Within a multi-layer context, the basic building units, namely neurons, are connected to each other among the adjacent layers via internal links, but the neurons belonging to the same layer have no connection, as depicted in Figure 1.

In Figure 1, each hidden layer processes its inputs via a connection function denoted as below,

(1) $$\begin{array}{c} h_{W,b}(X)=f(W^{T}X+b) \end{array}$$

where *W* refers to the weight and *b* for bias. When all input layer neurons are active, each input neuron will multiply their respective weight matrix and the output will be summed up with a bias, which then will be fed into an adjacent hidden layer. Although the input-output formalization may repeat similarly among hidden layers, there is usually no direct connection between neurons within the same layer. And activation function is to quantify the connection between two neighboring neurons across two (hidden) layers.

Specifically, the input of the activation function is the combination *W*^*T*^*X*+*b* denoted in Equation (1), and the function output is then fed into the next neuron as a new input. Following the connection formula, the former input feature can be extracted to the next layer; by this means the features can be well-extracted and refined further. And the performance of the feature extraction depends significantly on the selection of the activation function.

**Figure 2:**
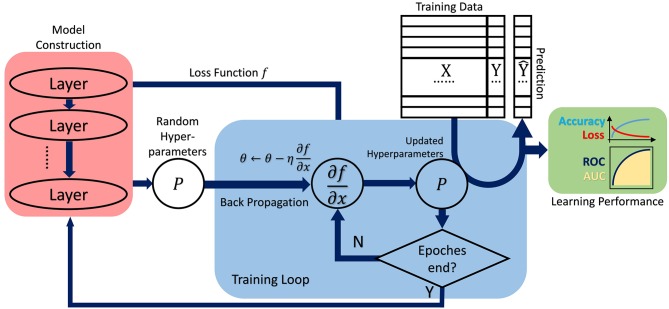
The general analysis procedure commonly adopted in deep learning, which covers training data preparation, model construction, hyperparameter fine-tuning (in training loop), prediction and performance evaluation. Basically, it still follows the requisite schema in machine learning.

Before training the network structure, the input raw datasets are usually separated into two or three groups, namely a training set and a test set, sometimes a validation set to examine the performance of previously trained network models, as depicted in Figure 2. In practice, the original datasets are separated stochastically to avoid the potential local tendency, but the proportion of each set can be determined manually.

### Learning by Training, Validation, and Testing

Normally, training a neural network refers to a process the network self-tunes its parameters or weights to meet the prespecified performance criteria, thus the trained model can be further used in regression or classification purposes. As depicted in Figure 2, generally a complete dataset collected from a specific experiment beforehand can be split into the training and testing, and even validation sets, then followed by conventional tasks as model training, validation and performance comparison.

During training with initial batches of data samples, model parameters and their characteristics normally can be tuned by various learning paradigms, including appropriate activation and rectification functions. Then the trained network should be further tested or even validated with the other batch of samples, to acquire high robustness and satisfactory predictability, the processes of which are often referred as model testing and validation.

Usually, the three procedures above are faithfully implemented in conventional machine learning studies; and even in its quickly-evolving subfield, deep learning, the similar paradigm is always observed (LeCun et al., 2015; Schmidhuber, 2015).

### Activation and Loss Function

After training completed, the neural network can perform regression or classification task on testing data, while there usually exists the difference between the predicted outputs and actual values. And the difference should be minimized to acquire optimal model performance.

Within a certain layer, error reduction requires scaling it back within a preset range before passing it onto the next layer of neurons. Activation herein is defined to control neurons' outputs in “active” or “inactive” status, using those non-linear functions as rectified linear unit (ReLU), tanh, and logistic (Sigmoid or soft step) (LeCun et al., 2015).

Besides, a loss function herein is to measure the total difference between the predicted and accurate values, through fine-tuning in backpropagation process. And it acts as an ending threshold for parameter optimization by means of iteratively evaluating the trained models.

With activation function in each neuron throughout diverse layers, a training procedure will continue searching a whole hyperparameter space till the ending threshold, compare and detect an optimal parameter combination by minimizing the preset loss function.

## Typical Algorithms and Applications

With the substantial progresses in advanced computation and Graphic Processing Unit (GPU) technologies, systematic interrogation into massive data to understand its inherent mechanisms becomes possible, especially through deep learning approaches. Hereinafter, we illustrated several frequently utilized models in deep learning literatures, in both recent computation theories and diverse applications.

### Recurrent Neural Network

Recurrent Neural Network (RNN) is a deep learning model different from traditional neural networks, since the former can integrate the previously learned status through a recurrent approach, namely backpropagation; while traditional neural network usually outputs prediction based on the status of the current layer.

Compared with traditional network models, RNN only has one hidden layer but it can unfold horizontally, and multi-vertical-groups are enabled to utilize most of the previous results, namely “using memory”.

**Figure 3:**
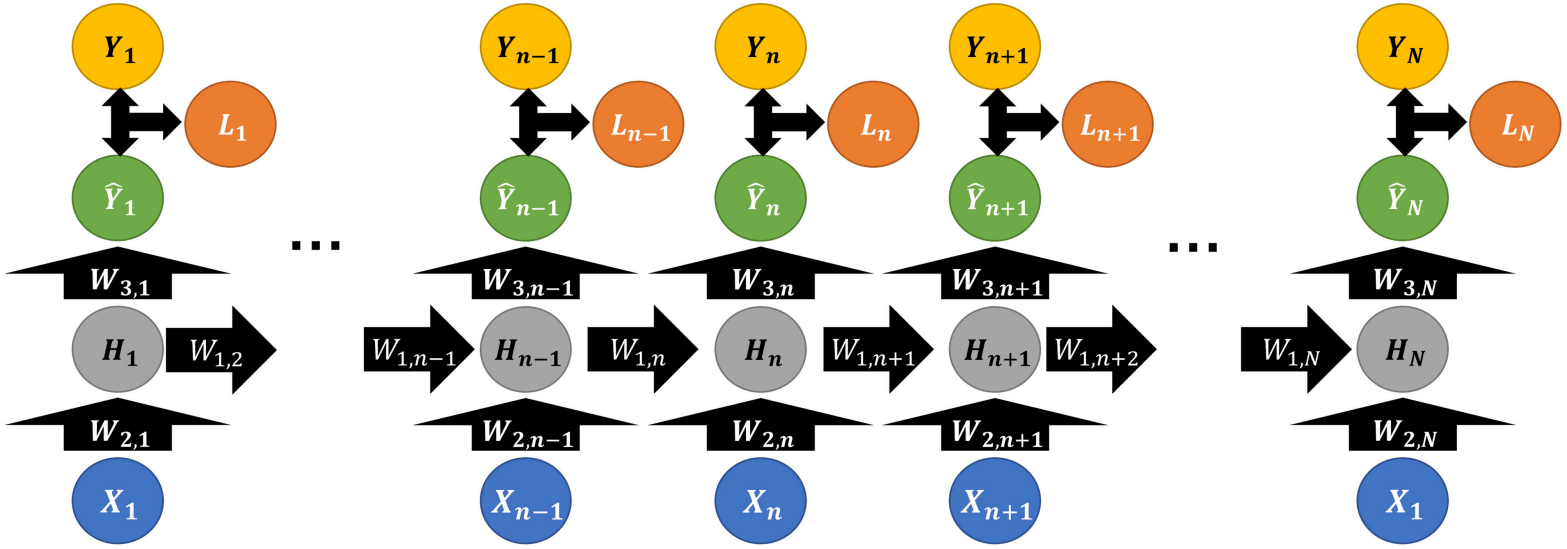
Illustrative structure diagram of Recurrent Neural Network, where *X, Y*, and *W* are defined the same as above; *L*_*i*_ denotes the loss function between the actual *Y*_*i*_ and predicted Ŷ_*i*_ (*i* ∈ *N*).

As depicted in Figure 3, the hidden layer neuron *H*_*n*_ is defined by Equation (2),

(2) $$\begin{array}{c} H_{n}=\sigma_{1}(W_{1,n}^{T}H_{n-1}+W_{2,n}^{T}X_{n}+b_{1,n}) \end{array}$$

where *W*_1, n_ and *W*_2, n_ represent weight matrix, *b*_1, n_ is a bias matrix, and σ(·) (usually *tanh*(·)) for an activation function. Thus, each layer will generate a partial of output from the current hidden layer neuron with a weight matrix *W*_3, n_ and bias *b*_2, n_, defined by Equation (3),

(3) $$\begin{array}{c} {\hat{Y}}_{n}=\sigma_{2}(W_{3,n}H_{n}+b_{2,n}) \end{array}$$

And the total loss *L*_*total*_ will be the sum of the loss functions from each hidden layer, defined as below,

(4) $$\begin{array}{c} L_{total}=\sum_{n=1}^{N}L_{n}=\sum_{n=1}^{N}L(\hat{Y},Y) \end{array}$$

Thus, fine tuning of RNN backpropagation is based on three weights, *W*_1, n_, *W*_2, n_, and *W*_3, n_. Since the multi-parameter setting in weights adds to the optimization burden, RNN usually performs worse than Convolutional Neural Network (CNN) in terms of fine-tuning. But frequently it is ensembled with CNN in diverse applications, such as dimension reduction, image, and video processing (Hinton and Salakhutdinov, 2006; Hu and Lu, 2018). Angermueller et al. proposed an ensembled RNN-CNN architecture, DeepCpG, on single-cell DNA methylation data, to better predict missing CpG status for genome-wide analysis; together the model's interpretable parameters shed light on the connection between sequence composition and methylation variability (Angermueller et al., 2017). Section Autoencoder will specifically discuss CNN and its typical applications.

Moreover, RNN outperforms those conventional models as logistic regression and SVM, and it can be implemented in various environments, accelerated by GPUs (Li et al., 2017). Due to its structural characteristics, RNN is suitable to deal with long and sequential data, such as DNA array and genomics sequence (Pan et al., 2008; Ray et al., 2009; Jolma et al., 2013; Lee and Young, 2013; Alipanahi et al., 2015; Xu T. et al., 2016).

But RNN cannot interact with hidden neurons far from the current one. To construct an efficient framework of recalling deep memory, many improved algorithms have been proposed, like BRNN in protein secondary structure prediction (Baldi et al., 1999), and MD-RNN in analyzing electron microscopy and MRIs of breast cancer samples (Kim et al., 2018).

**Figure 4:**
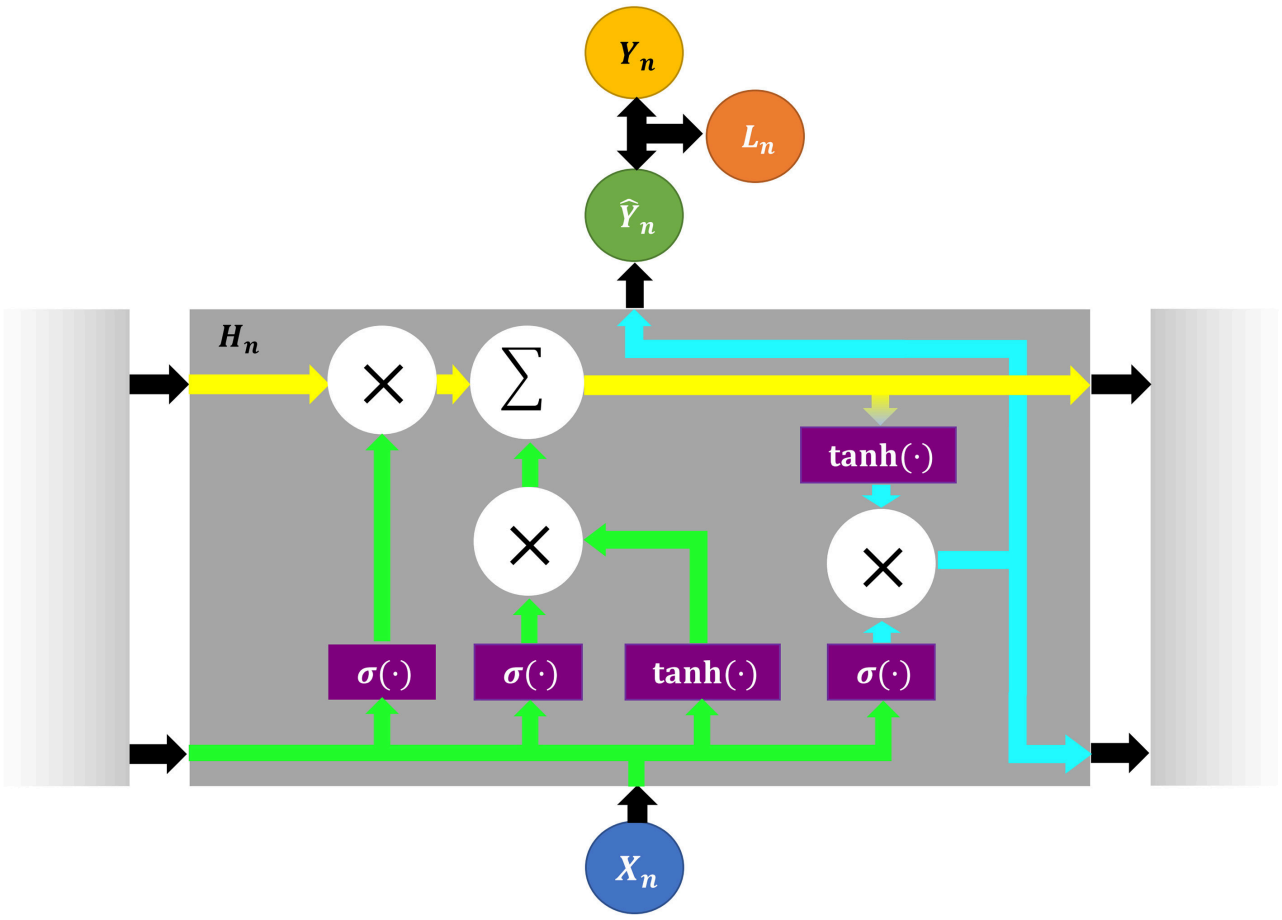
The LSTM network structure and its general information flow chart, where *X, Y*, and *W* are defined the same as above.

LSTM (Long Short-Term Memory) and GRU (Gated Recurrent Unit) are two recently-improved derivatives of RNN to solve the long-time dependence issues. GRU shares a similar structure with LSTM, which has several gates used for modeling its memory center. The current memory output is jointly influenced by its current input feature, the context (namely the past influence), and the inner action toward the input, as shown in Figure 4.

In Figure 4, the yellow track refers to an input gate transfering its total past features, and is accessible for any new feature to be added. The green track is a mixture of an input gate and its former hidden layer neurons; and it decides what to omit, namely resetting activation function close to 0, and what to be updated into the yellow track. The blue track is the output gate integrating the inner influence from the yellow track, and it decides the output of the current hidden neurons and what to be passed to the next hidden neuron.

Recently an attention-based architecture, DeepDiff, utilizes a hierarchy of LSTM modules to characterize how various histone modifications cooperate simultaneously, and it can effectively predict cell-type-specific gene expression (Sekhon et al., 2018).

### Convolutional Neural Network

Convolutional neural networks (CNN or ConvNet) are suitable to process information in the form of multiple arrays (LeCun et al., 2015; Esteva et al., 2017; Hu and Lu, 2018). To reduce the parameters without compromising its learning capacity is the general design principle of CNN (LeCun et al., 2015; Krizhevsky et al., 2017). And each convolution kernel's parameters in CNN are trained by the backpropagation algorithm.

**Figure 5:**
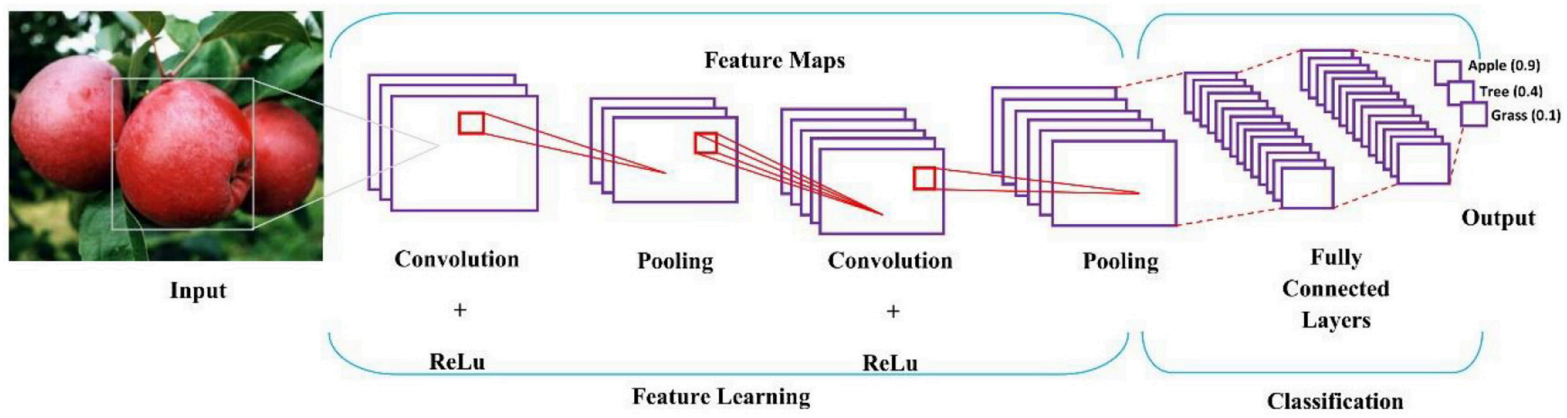
The basic architecture and analysis procedure of a CNN model, which illustrates a classification procedure for an apple on a tree.

Especially in image-related applications, CNN can cope with pixel scanning and processing, thus it greatly accelerates the implementation of optimized algorithms into practice (Esteva et al., 2017; Quang et al., 2018). Structurally, CNN consists of linear convolution operation, followed by nonlinear activators, pooling layers, and deep neural network classifier, depicted in Figure 5.

In Figure 5, several filters are applied to convolve an input image, and its output is subsampled as a new input into the next layer; and convolution and subsampling processes are repeated till high level features, namely shapes, can be extracted. The more layers a CNN model has, the higher-level features it will extract.

In feature learning, convolution operation is to scan a 2D image with a given pattern, and calculate the matching degree at each step, then pooling identifies the pattern presence in the scanned region (Angermueller et al., 2016). Activation function defines a neuron's output based on a set of given inputs. The weighted sum of inputs is passed through an activation function for non-linear transformation. A typical activation function returns a binary output, 0 or 1; when a neuron's accumulation exceeds a preset threshold, the neuron is activated and passes its information to the next layers; otherwise, the neuron is deactivated. Sigmoid, tanh, ReLU, leaky ReLU, and softmax are the commonly used activation functions (LeCun et al., 2015; Schmidhuber, 2015).

Through pooling layers, pixels are stretched to a single column vector. The vectorized and concatenated pixel information is fed into dense layers, known as fully connected layers for further classification. The fully-connected layer renders the final decision, where CNN returns a probability that an object in the image belongs to a specific type.

Following the fully-connected layer is a loss layer, which adjusts their weights across the network. A loss function is used to measure the model performance and inconsistency between the actual and predicted values. Model performance increases with decreasing of the loss function. For an output vector *y*_*i*_ and an input *x* = (*x*_1_, *x*_2_, …, *x*_*n*_), the mapping loss function *L*(·) between *x* and *y* is defined as,

(5) $$\begin{array}{c} L(y_{i},{\hat{y}}_{i})=\frac{1}{n}\sum_{i=1,j=1}^{n,k}\varphi [y_{i},f(x_{i},\sigma_{i},\omega_{ij},b_{i})] \end{array}$$

where φ denotes an empirical risk for each output, ŷ_*i*_ for the *i*-th prediction, *n* the total number of training samples, *k* the count of the weights ω_*ij*_ and *b*_*i*_ the bias for the activation function σ_*i*_.

Recently, CNN has been adopted rapidly in biomedical imaging studies for its outstanding performance in computer vision and concurrent computation with GPUs (Ravi et al., 2017). Usually convolution-pooling structure can better learn imaging features from CT scans and MRI images from head trauma, stroke diagnosis and brain EPV (enlarged perivascular space) detection (Chilamkurthy et al., 2018; Dubost et al., 2019).

In recent computational biology, a discriminative CNN framework, DeepChrome, is proposed to predict gene expression by feature extraction from histone modification. And the deep learning model outperforms traditional Random Forests and SVM on 56 cell types from REMC database (Singh et al., 2016).

Furthermore, CNN can be combined with other deep learning models, such as RNN to predict imaging content, where CNN encodes an image and RNN generates the corresponding image description (Angermueller et al., 2016). Till now, quite a few variants of CNN have been also proposed in diverse classification applications, like AlexNet with GPU support and DQN in reinforcement learning (Mnih et al., 2015).

### Autoencoder

Through an unsupervised manner, autoencoder is another typical artificial neural network, designed to precisely extract coding or representation features using data-driven learning (Min et al., 2017; Zeng et al., 2017; Yang et al., 2018). For high-dimensional data, it is time-consuming and infeasible to load all raw data into a network, thus dimension reduction or compression is a necessity in preprocessing of raw data.

**Figure 6:**
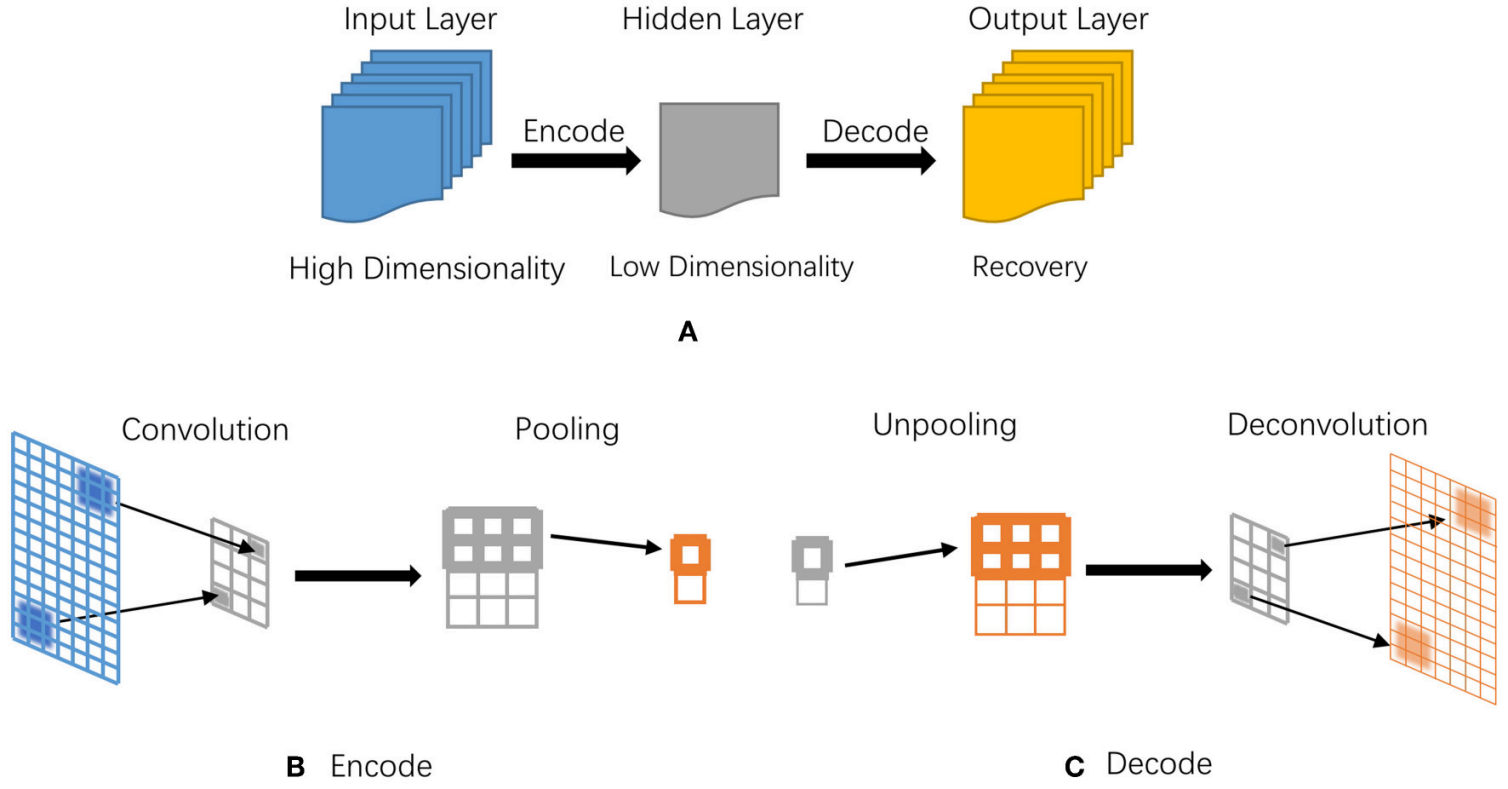
The illustrative diagram of an autoencoder model. **(A)** Basic processing structure of autoencoder, corresponding to the input, hidden, and output layers; **(B)** Processing steps in encoding; **(C)** Processing steps in decoding.

Autoencoder can compress and encode information from the input layer into a short code, then after specific processing, it will decode into the output closely matching the original input. Figure 6 illustrates its basic model structure and processing steps.

Convolution and pooling are two major steps in encoder, depcited in Figure 6B; while decoder has two complete opposite steps, namely unpooling and deconvolution in Figure 6C. Both convolution and pooling can compress data while preserving the most representative features in two different ways. Convolution involves continuously scanning data with a rectangle window, for example a 3 × 3 size; after each scanning, the window moves to a next position, namely pixel, by replacing the oldest elements with new ones, together with convolution operation. After the whole scanning and convolution, pooling is utilized to deeper compress on redundancy.

Similar to traditional PCA in dimension reduction to some extent, but autoencoder is more robust and effective in extracting data features for its non-linear transformation in hidden layers. Given an input *x*, the model extracts its main feature and generates $\hat{x}=Wb$, where *W* and *b* denote weighting and bias vectors, respectively. Commonly, the output cannot fit the input precisely, which can be measured with a loss function in mean squared error (MSE) defined in Equation (6),

(6) $$\begin{array}{c} L(W,b)=\frac{1}{m}\sum_{i=1}^{m}{\left(\hat{x}-x\right)}^{2} \end{array}$$

Thus, the learning process is to minimize the loss *L* after iterative optimization.

Recently, sparse autoencoder (SAE) is frequented discussed for its admirable performance in dimension reduction and denoising corrupted data. And the loss function in SAE is defined in Equation (7),

(7) $$\begin{array}{c} L_{SAE}=L(W,b)+\beta \sum_{k}KL(\rho ∥\hat{\rho_{k}}) \end{array}$$

where KL refers to KL-divergence in Equation (10), ρ for the activation level of neurons, usually set as 0.05 in condition of sigmoid, indicating most neurons are inactive, ρ_*k*_ for the average activation level of neuron *k*, and β for the regularization coefficient.

(8) $$KL(\rho ∥\;\hat{\rho_{k}}\text{)}=\rho log\frac{\rho }{\hat{\rho_{k}}}+(1-\rho )log\frac{1-\rho }{1-\hat{\rho_{k}}}$$

where $\hat{\rho_{k}}$ represents the average activation level of test samples, and *x*^(*i*)^ is the *i*-th test sample in Equation (9).

(9) $$\begin{array}{c} \hat{\rho_{k}}=\frac{1}{m}\sum_{i}\left[a_{j}(x^{\left(i\right)})\right] \end{array}$$

For high dimensional data, multiple autoencoders can be stacked to act as a deep autoencoder (Hinton and Salakhutdinov, 2006). And this architecture may lead to vanishing gradient, due to its gradient-based and backpropagation learning, and the current solutions include adopting ReLu activation and dropout (Szegedy et al., 2015; Krizhevsky et al., 2017). During configuration and pretraining, the model weights can be acquired by greedy layer-wise training, then the network can be fine-tuned with the backpropagation algorithm.

Many variations of autoencoder have been proposed recently, such as sparse autoencoder (SAE), denoising autoencoder (DAE). Typically, stacked sparse autoencoder (SSAE) was proposed to analyze high-resolution histopathological images in breast cancer (Xu J. et al., 2016). By using SAE with three iterations, Heffernan et al. reported the successful prediction of protein secondary structure, local backbone angles, and solvent accessible surface area (Heffernan et al., 2015). Miotto et al. introduced a stack of DAEs to predict features from a large scale of electronic health records (EHR), via an unsupervised representation approach (Miotto et al., 2016). Ithapu et al. proposed a randomized denoising autoencoder marker (rDAm) to predict future cognitive and neural decline for Alzheimer diseases, with its performance surpassing the existing methods (Ithapu et al., 2015).

### Deep Belief Network

**Figure 7:**
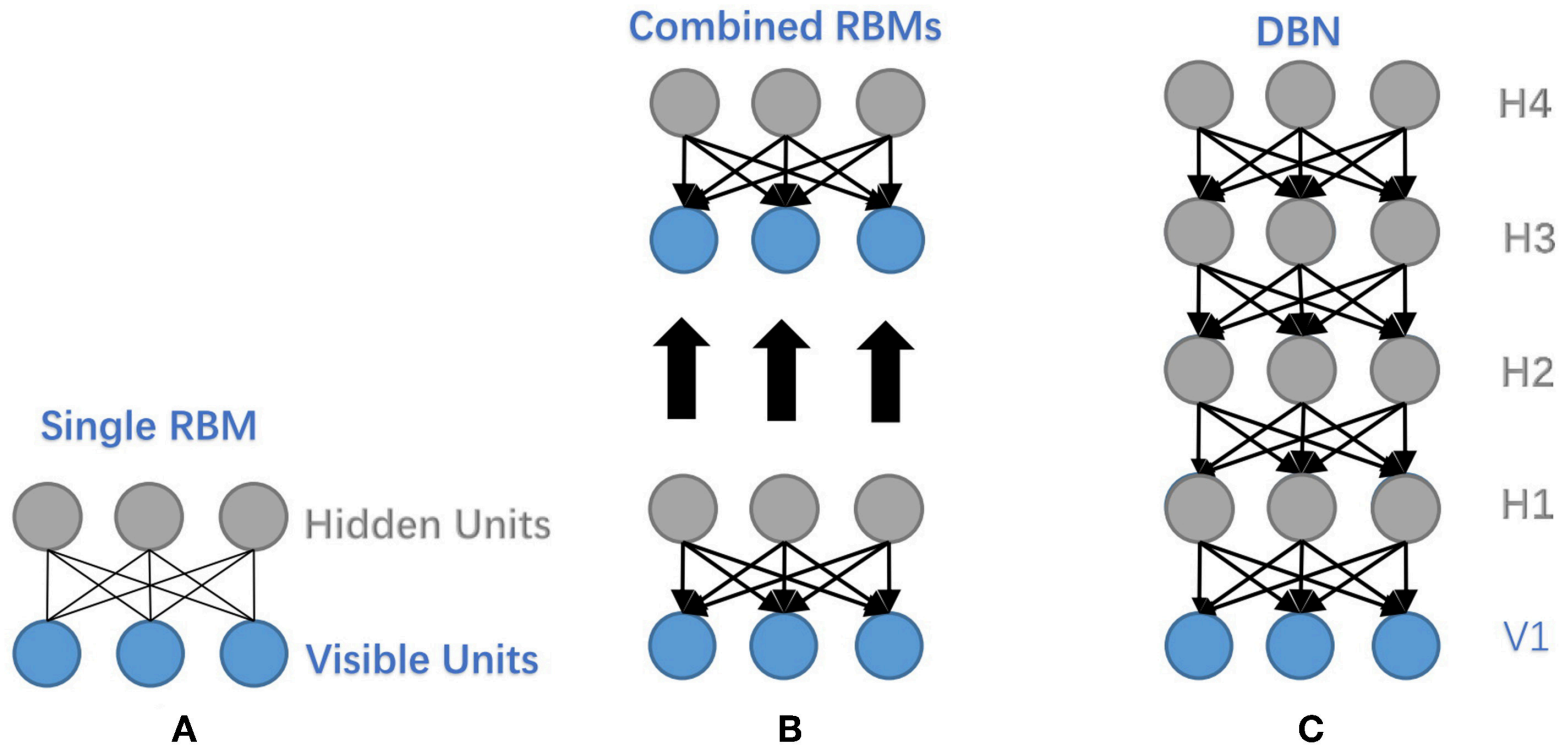
Illustrative network structures of RBM and DBN. **(A)** The structure of RBM. **(B)**Take the hidden layer of the trained RBM to function as the visible layer of another RBM. **(C)** The structure of a DBN. It stacks several RBMs on top of each other to form a DBN.

As a generative graphical model, Deep Belief Network (DBN) is composed of multiple Restricted Boltzmann Machines (RBM) or autoencoders stacked on top of each other, where each hidden layer in subnetworks serves as a visible layer for the next layer (Hinton et al., 2006). The main network structures of RBM and DBN are depicted in Figure 7, where it manifests the construction relations between the two network models.

DBN trains layer by layer in an unsupervised greedy approach to initialize network weights, separately; then it can utilize the wake-sleep or backpropagation algorithm during fine-tuning. While for traditional backpropagation used in fine-tuning, DBN may encounter several problems: (1) requiring labeled data for training; (2) low learning rate; (3) inappropriate parameters tending to acquire local optimum.

Within recent applications, Plis et al. classified schizophrenia patients based on brain MRIs with DBN (Plis et al., 2014); in drug design based on high-throughput screening, DBN was exploited to perform quantitative structure activity relationship (QSAR) study. And the result showed that the optimization in parameter initialization highly improves the capability of DNN to provide high-quality model predictions (Ghasemi et al., 2018). DBN was also used to study the combination of resting-state fMRI (rs-fMRI), gray matter, and white matter data by exploiting the latent and abstract high-level features (Akhavan Aghdam et al., 2018). Meanwhile, DBN and CNN were compared to prove that deep learning has better discriminative results and holds promise in the medical image diagnosis (Hua et al., 2015).

### Transfer Learning in Deep Learning

Besides the above deep learning models, transfer learning is frequently utilized in specific cases without sufficient labeling information or dimensionality (Pan and Yang, 2010). Although conceptually it does not belong to deep learning, due to its transferability of high-level semantic classification for deep neural network, transfer learning has gained emerging notices from deep learning fields (O'Shea et al., 2013; Anthimopoulos et al., 2016).

**Figure 8:**
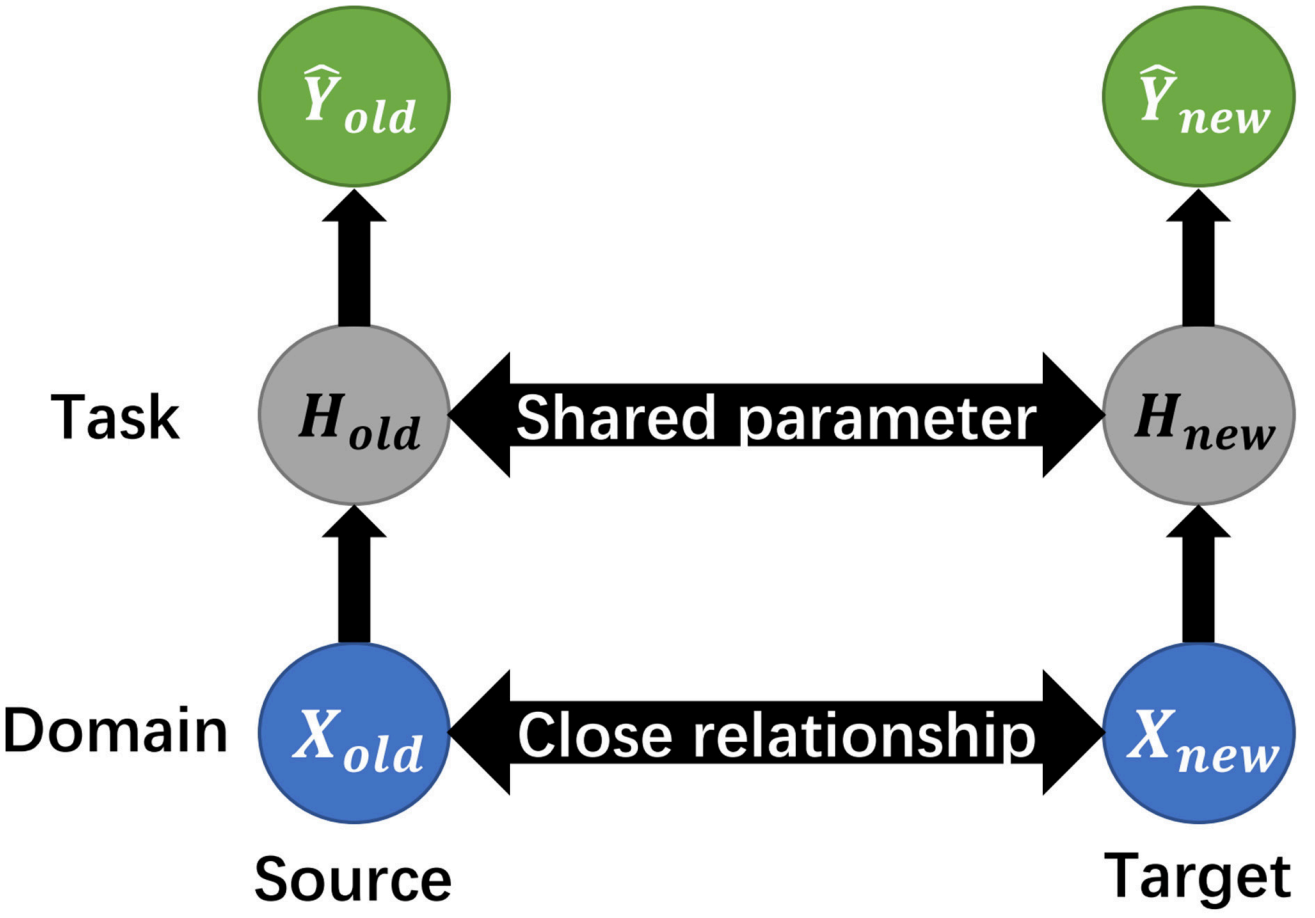
The schematic illustration of transfer learning. Given source domain and its learning task, together with target domain and respective task, transfer learning aims to improve the learning of the target prediction function, with the knowledge in source domain and its task.

In quite a few deep learning studies, transfer learning enables a previously-trained model to transfer its optimized parameters to a new model, thus to implement the knowledge transmission and reduce repetitive training from scratch, as depicted in Figure 8.

Normally, source and target domains have certain statistical relationship or similarity that directly affects the transferability. The domain contains the original dataset, for example image matrix, and the task refers to certain processes, like classification or pattern recognition. The mission of transfer learning includes transferring not only the parameters like weight, but the concentrated small-size matrix from the origin data domain called knowledge distillation.

The knowledge distillation usually uses both “hard target” and “soft target” to train the model and obtain lower information entropy. The below softmax function is usually utilized to soften the sparse data and excavate its inherent features,

(10) $$f(\alpha_{k})=\frac{e^{\frac{\alpha_{k}}{T}}}{\sum_{k}e^{\frac{\alpha_{k}}{T}}}$$

where the logical judger α_*k*_ is the input, *f* (·) is to soft target data and can offer smaller gradient variance, *k* denotes the *k*-th segmented data slice. The parameter *T* is called temperature and the larger *T* is, the softer the target is.

**Figure 9:**
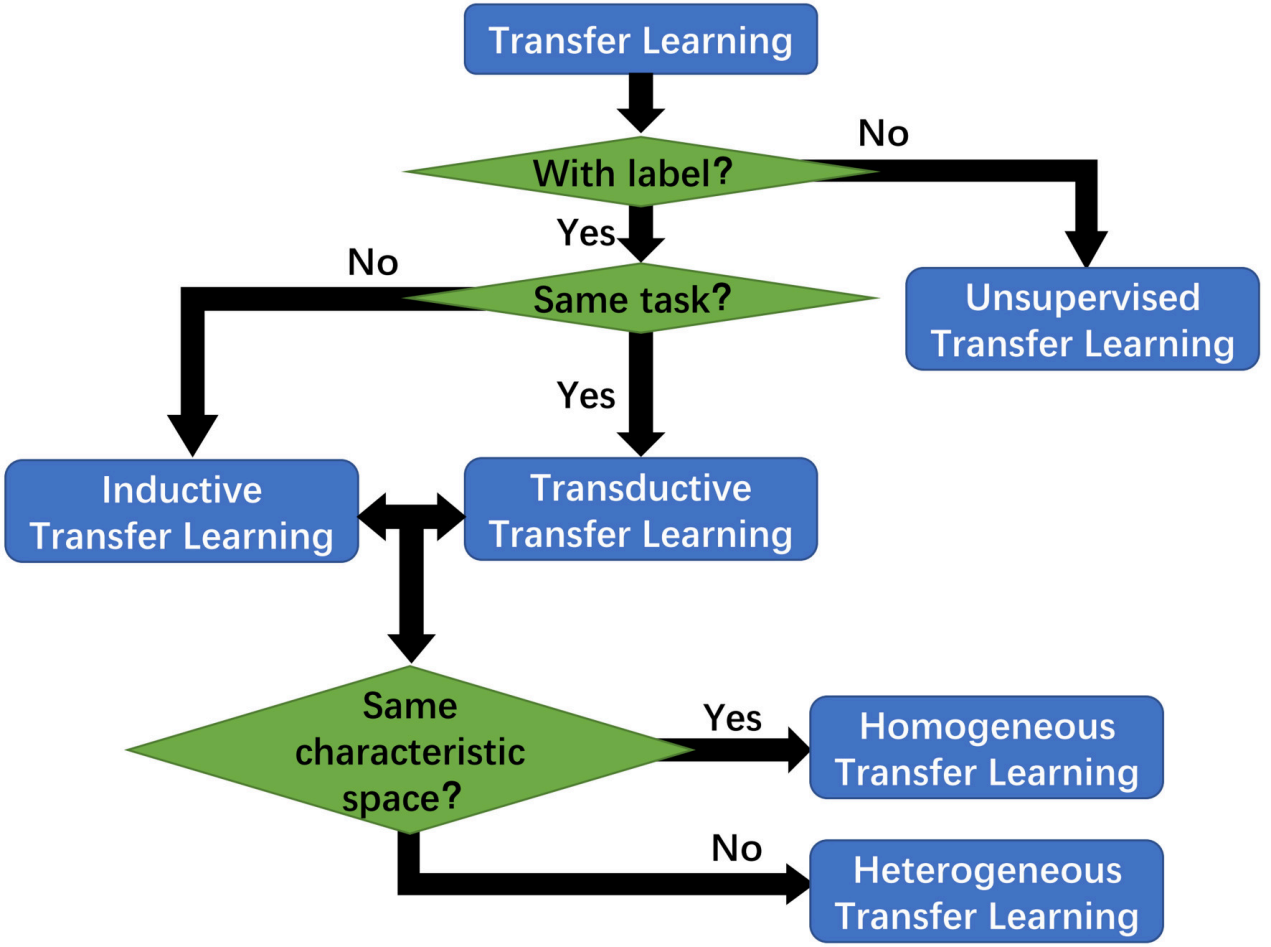
Transfer learning has several derivatives categorized by the labeling information and difference between the target and source.

Furthermore, transfer learning is categorized into instance-based, feature-based, parameter-based and relation-based derivatives, depicted in Figure 9. Currently transfer learning is frequently discussed in the deep learning fields for its great applicability and performance. Ensembled with CNN, transfer learning can attain greater prediction performance of interstitial lung disease CT scans (Anthimopoulos et al., 2016). It was also used as a ligament between the multi-layer LSTM and conditional random field (CRF), and the result showed that the LSTM-CRF approach outperformed the baseline methods on the target datasets (Giorgi and Bader, 2018).

## Conclusions

Within the work, we comprehensively summarized the basic but essential concepts and methods in deep learning, together with its recent applications in diverse biomedical studies. Through reviewing those typical deep learning models as RNN, CNN, autoencoder, and DBN, we highlight that the specific application scenario or context, such as data feature and model applicability, are the prominent factors in designing a suitable deep learning approach to extract knowledge from data; thus, how to decipher and characterize data feature is not a trivial work in deep-learning workflow yet. In recent deep learning studies, many derivatives from classic network models, including the network models depicted above, manifest that model selection affects the effectiveness of deep learning application.

Secondly, for its limitation and further improvement direction, we should revisit the nature of the method: deep learning is essentially a continuous manifold transformation among diverse vector spaces, but there exist quite a few tasks cannot be converted into a deep learning model, or in a learnable approach, due to the complex geometric transform. Moreover, deep learning is generally a big-data-driven technique, which has made it unique from conventional statistical learning or Bayesian approaches. Thus, it is a new direction for deep learning to integrate or embed with other conventional algorithms in tackling those complicated tasks.

Thirdly, when it comes to innovation in computational algorithm and hardware. As an inference technique driven by big data, deep learning demands parallel computation facilities of high performance, together with more algorithmic breakthroughs and fast accumulation of diverse perceptual data, it is achieving pervasive successes in many fields and applications. Particularly in bioinformatics and computational biology, which is a typical data-oriented field, it has witnessed the remarkable changes taken place in its research methods.

Finally, as unprecedented innovation and successes acquired with deep learning in diverse subfields, some even argued that deep learning could bring about another wave like the internet. In the long term, deep learning technique is shaping the future of our lives and societies to its full extent. But deep learning should not be misinterpreted or overestimated either in academia or AI industry, and actually it has lots of technical problems to solve due to its nature. In all, we anticipate this review work will provide a meaningful perspective to help our researchers gain comprehensive knowledge and make more progresses in this ever-faster developing field.

## Author Contributions

BT conceived the study. ZP, KY, AK, and BT drafted the application sections and revised and approved the final manuscript.

### Conflict of Interest Statement

The authors declare that the research was conducted in the absence of any commercial or financial relationships that could be construed as a potential conflict of interest.
